# Oleum cinnamomi disrupts *Cutibacterium acnes* adhesion and metabolism by covalently targeting enolase and coenzyme A

**DOI:** 10.3389/fmicb.2026.1919350

**Published:** 2026-09-04

**Authors:** Jianchao Wang, Lanting Zhou, Xiaonuo Zhong, Yingchao Wang, Huanjing Wang, Xinjun Xu, Huayong Peng, Depo Yang

**Affiliations:** 1 Key Laboratory of Medicinal Resources Chemistry and Pharmacology in Wuling Mountainous of Hunan Province College, Jishou University, Jishou, China; 2 School of Pharmaceutical Sciences, Sun Yat-sen University, Guangzhou, China

**Keywords:** adhesion, coenzyme A, covalent inhibitors, enolase, oleum cinnamomi, sugar metabolism

## Abstract

**Background:**

Preventing *Cutibacterium acnes* (*C. acnes*) adhesion to human skin is essential for acne control, yet the molecular determinants of adhesion-associated drug resistance remain undefined and no pharmacological agents specifically blocking *C. acnes* attachment are available.

**Objective:**

To evaluate oleum cinnamomi (OC), a natural cinnamon oil, as a potential anti-adhesive modality against *C. acnes*.

**Methods:**

*In vitro* antibacterial assays, quantitative chemoproteomics, and keratinocyte infection models were integrated to quantify OC-mediated inhibition of *C. acnes* adhesion and biofilm formation, map covalent targets of OC electrophiles cinnamaldehyde (CA) and 2-methoxycinnamaldehyde (MCA), and delineate downstream metabolic perturbations via untargeted metabolomics and redox profiling.

**Results:**

OC, CA and MCA concentration-dependently reduced *C. acnes* adhesion (> 80%) without rapid bactericidal activity. Both CA and MCA covalently modified Cys359 of enolase (ENO), suppressing glycolytic flux and ATP generation. Independently, CA formed a thiohemiacetal adduct with coenzyme A (CoA), blocking acyl-transfer reactions required for fatty-acid metabolism and membrane biogenesis. The combined effects elevated the NADH/NAD^+^ ratio, disrupted oligosaccharide homeostasis, and impaired biofilm exopolysaccharide biosynthesis with altered monosaccharide composition, thereby compromising biofilm formation and driving *C. acnes* into a metabolically compromised state.

**Conclusion:**

OC-derived electrophiles act as metabolic modulators that incapacitate *C. acnes* adhesion and biofilm development through orthogonal inhibition of ENO and CoA-dependent pathways, providing a mechanistic blueprint for potential topical anti-acne therapies that circumvent conventional antibiotic resistance.

## 1 Introduction

*Cutibacterium acnes* (*C. acnes*), a Gram-positive, anaerobic commensal that dominates the sebaceous follicle, is now recognized as a key pathogen driving acne vulgaris (Feng et al., 2024; Norton et al., 2012). By secreting lipases and proteases that hydrolyze sebum triglycerides, the bacterium generates free fatty acids and bioactive peptides that rupture the follicular wall and incite local inflammation (Kmieciak and Szewczyk, 2018). Concurrently, *C. acnes* triggers monocytes and keratinocytes to release IL-1β, TNF-α and other cytokines, thereby amplifying lesion progression (Feng et al., 2024). Of greater concern, decades of prolonged antibiotic therapy have selected for tetracycline- and macrolide-resistant clones worldwide (Dessinioti and Katsambas, 2024; Dréno et al., 2018); tetracycline resistance has risen > 100-fold, and cross-resistance in staphylococci has been documented (Legiawati et al., 2023). Consequently, there is an urgent unmet need for anti-acne agents that operate through novel, resistance-averse mechanisms.

Cutaneous colonization is the obligate first step in *C. acnes* pathogenesis, and adherence to host cells and sebaceous lipids is the decisive event that enables persistence (Cavallo et al., 2022; Thoraval et al., 2025). Although electron micrographs hint at surface appendages (fimbriae, pili or outer membrane adhesins) that might engage glycoprotein/glycolipid receptors on keratinocytes and sebocytes, the specific adhesins and their cognate host ligands remain unidentified (Kaplan et al., 2024; Mayslich et al., 2021). To date, no small-molecule or biological inhibitor that selectively blocks *C. acnes* adhesion has been characterized, leaving a critical gap in our therapeutic arsenal.

Oleum cinnamomi (OC), the essential oil distilled from *Cinnamomum cassia*, is rich in cinnamaldehyde (CA) and 2-methoxycinnamaldehyde (MCA)—α,β-unsaturated carbonyls that function as privileged electrophilic “warheads” (Bampidis et al., 2022; Rao and Gan, 2014). Having been used for centuries as a food flavoring and traditional medicine, OC exhibits an established safety profile in humans and animals (Peng et al., 2024; Vasconcelos et al., 2018). Independently, OC and its constituents have been reported to possess antibacterial, anti-inflammatory and anti-biofilm activities. α,β-unsaturated aldehydes are known to undergo Michael addition with cysteine thiols, irreversibly modifying target proteins and abrogating their function (Alibi et al., 2022; Wultańska et al., 2024).

Building on these precedents, we hypothesized that OC blocks *C. acnes* colonization by covalently modifying key adhesins and/or metabolic enzymes essential for adhesion. Here we integrate quantitative antibiograms, keratinocyte infection models, chemical proteomics, untargeted metabolomics and biofilm imaging to delineate the mechanism by which OC and its major components CA and MCA inhibit *C. acnes* adhesion. We demonstrate that CA and MCA covalently labels Cys359 of enolase (ENO), impairing the glycolytic conversion of 2-phosphoglycerate to phosphoenolpyruvate. Concurrently, the aldehydes form stable thiohemiacetals with coenzyme A (CoA), disrupting intracellular acyl-transfer reactions. The cumulative outcome is a rewired sugar metabolome, an elevated NADH/NAD^+^ ratio, reduced ATP levels, impaired biosynthesis of biofilm exopolysaccharides (B-EPS) with altered monosaccharide composition, and ultimately attenuated bacterial adhesion and growth arrest. This study provides the first target–metabolism–phenotype blueprint explaining how OC disrupts *C. acnes* adhesion, and offers a naturally derived scaffold for potential anti-acne agents that act upstream of inflammation by preventing pathobiont colonization.

## 2 Materials and methods

### 2.1 Materials and reagents

*C. acnes* ATCC 6919 was obtained from the Guangdong Microbial Culture Collection Center (Guangzhou, China). HaCaT cells (passages 3–10) were purchased from the Cell Bank of the Chinese Academy of Sciences (Shanghai, China). Brain heart infusion (BHI) broth was from HuanKai Microbial (Guangdong, China). Cinnamaldehyde (CA, ≥ 98% purity), 2-methoxycinnamaldehyde (MCA, ≥ 98%), trypsin, and HPLC-grade formic acid were from Sigma-Aldrich. Coenzyme A (CoA), NAD^+^/NADH assay kits, and enhanced BCA protein assay kits were from Beijing Solarbio Science & Technology. CFDA-SE was from Med Chem Express. All other reagents were analytical grade.

### 2.2 Preparation and GC-MS analysis of OC

Fresh *Cinnamomum cassia* bark was collected in Luoding County, Guangdong, China, and authenticated by Prof. Yang Depo, Sun Yat-sen University (voucher no. 20230125004, SYU Medicinal Herbarium). Essential oil was isolated by steam distillation (100 g powdered bark, 20-mesh [0.85 mm] sieve, in 900 mL deionized water) for 3 h. The oil was dried over anhydrous Na_2_SO_4_ and filtered.

For GC-MS analysis, OC was dissolved in n-hexane (1:200, v/v). A 1-μL aliquot was injected into a Thermo Trace DSQ II system equipped with a DB-5MS capillary column (30 m × 0.25 mm × 0.25 μm; Agilent). Helium carrier gas flow was 1 mL/min. Oven temperature program: 50 °C (2 min), ramped to 220 °C at 5 °C/min, held for 15 min. MS parameters: EI 70 eV, m/z 35–300. Components were identified by matching retention indices (RI) and mass spectra against the NIST 2017 library (Namkona et al., 2024).

### 2.3 Antimicrobial susceptibility testing

MIC and MBC were determined by broth microdilution according to CLSI guidelines (Chen J. et al., 2022), with modifications for anaerobic conditions. Stock solutions of OC, CA, MCA, and analogs were prepared in 20% (v/v) DMSO at 200 mg/mL and serially diluted two-fold in BHI to final concentrations of 1–1,000 μg/mL (final DMSO ≤ 0.1%). Tetracycline served as positive control. *C. acnes* (∼5 × 10^5^ CFU/mL) was inoculated into 96-well plates and incubated anaerobically (AnaeroPack, Mitsubishi Gas Chemical) at 37 °C for 48 h. MIC was the lowest concentration inhibiting visible growth. For MBC, 100 μL from wells showing no growth was plated on BHI agar and incubated anaerobically for 72 h; MBC was the lowest concentration yielding ≥ 99.9% reduction in CFU.

### 2.4 Adhesion assays

#### 2.4.1 Bacterial pre-treatment and CFDA-SE labeling

*C. acnes* in logarithmic phase was resuspended in fresh BHI to ∼1.0 × 10^8^ CFU/mL. Bacterial suspensions were pre-treated with OC (55 or 110 μg/mL), CA (50 or 100 μg/mL), or 0.1% DMSO (vehicle) under anaerobic conditions at 37 °C with shaking (180 rpm) for 8 h. Pre-treated bacteria were harvested by centrifugation (4,000 × *g*, 6 min, 4 °C), washed twice with ice-cold PBS, and resuspended in PBS to OD_600_ = 0.2. For fluorescence labeling, bacteria were incubated with 2 μM CFDA-SE at 37 °C for 20 min in the dark, washed three times with HBSS containing 1% FBS, and resuspended to 1 mL. Labeled bacteria were used within 2 h (Garcia et al., 2021).

#### 2.4.2 Qualitative assessment by integrated cell imaging

HaCaT cells were seeded at 5 × 10^5^ cells/well in 12-well plates and cultured in DMEM (10% FBS, 1% penicillin-streptomycin) at 37 °C, 5% CO_2_ for 24 h to confluence, then matured for an additional 24 h without antibiotics. Cells were washed twice with HBSS/1% FBS and infected with CFDA-SE-labeled *C. acnes* (MOI = 10) at 37 °C for 2 h. After three washes with HBSS/1% FBS, adhesion was visualized using an EVOS M700 imaging system (Thermo Scientific) within 1 h.

#### 2.4.3 Quantitative analysis by flow cytometry

HaCaT cells were plated in 6-well plates (1 × 10^6^ cells/well) and cultured as above. Cells were washed, trypsinized, and resuspended in HBSS/1% FBS. CFDA-SE-labeled *C. acnes* (MOI = 10) was added and incubated at 37 °C for 2 h. Cell suspensions were centrifuged (1,000 × *g*, 3 min, 4 °C), washed twice, resuspended in HBSS/1% FBS, and analyzed on a CytoFLEX S flow cytometer (Beckman Coulter). Uninfected HaCaT cells served as negative control. At least 10,000 events were recorded per sample. Adhesion rate was calculated as the percentage of CFDA-SE-positive cells.

### 2.5 Label-free quantitative proteomics

*C. acnes* was treated with 1 × MIC OC (55 μg/mL) or 0.1% DMSO under anaerobic conditions for 8 h. Cells were harvested (8,000 × g, 5 min), washed with ice-cold PBS, and lysed in SDT buffer (4% SDS, 100 mM DTT, 100 mM Tris-HCl, pH 8.0) at 95 °C for 5 min. Proteins were extracted by sonication (5 min, 3 s on/6 s off, 40% amplitude), clarified (8,000 × g, 10 min, 4 °C), and quantified by BCA assay.

For digestion, proteins were precipitated with ice-cold acetone (1:4, v/v) at −20 °C overnight, collected (8,000 × g, 30 min, 4 °C), washed with 80% acetone, and resuspended in UA buffer (8 M urea, 0.1 M Tris-HCl, pH 8.5). Reduction was performed with 10 mM DTT at 30 °C for 1.5 h, followed by alkylation with 50 mM iodoacetamide (IAA) in the dark at room temperature for 40 min. Samples were diluted 10-fold with 50 mM ammonium bicarbonate and digested with sequencing-grade trypsin (1:60 w/w, 37 °C, 12 h). Peptides were desalted on C18 StageTips and dried by vacuum centrifugation.

Peptides were separated on an EASY-nLC 1200 system (Thermo Scientific) equipped with a ReproSil-Pur C18-AQ column (75 μm × 30 cm, 2 μm; Dr. Maisch). Mobile phase A: 0.1% formic acid in water; B: 0.1% formic acid in 80% acetonitrile. Gradient: 5%–35% B over 90 min, 35%–95% B over 5 min, held at 95% B for 5 min; flow rate 200 nL/min.

MS analysis was performed on a Q Exactive Plus (Thermo Scientific) in data-dependent acquisition (DDA) mode. Full MS: 350–1,800 m/z, resolution 70,000, AGC target 3 × 10^6^, maximum IT 50 ms. dd-MS^2^: resolution 17,500, AGC target 1 × 10^5^, maximum IT 100 ms, NCE 27, isolation window 1.6 m/z, dynamic exclusion 30 s.

Database searching was performed with Proteome Discoverer 2.2 (Thermo Scientific) against the UniProt *C. acnes* database (downloaded 5 May 2024) using SEQUEST HT. Parameters: precursor mass tolerance 10 ppm; fragment mass tolerance 0.02 Da; fixed modification: carbamidomethylation (C); variable modifications: oxidation (M), acetylation (protein N-terminus). False discovery rate (FDR) was set to 1% at both peptide and protein levels (Tang et al., 2020).

### 2.6 Chemical proteomics for covalent target identification

For *in vitro* reactivity, CA (2.64 mg, 20 μmol) was dissolved in 20 μL DMSO and diluted with 180 μL PBS (pH 7.4). CoA (15.35 mg, 20 μmol) was added, and the mixture was incubated at 37 °C with shaking (300 rpm) for 2 h. The reaction was quenched with ice-cold acetonitrile (1:1, v/v), centrifuged (12,000 × *g*, 10 min), and the supernatant was analyzed directly.

For target identification *in vivo*, *C. acnes* was treated with 1 × MIC CA (50 μg/mL) or vehicle for 4 h. Cells were lysed in PBS containing 1% Triton X-100 and protease inhibitors. Lysates were denatured at 95 °C, reduced, alkylated, and digested as described in Section “2.5 Label-free quantitative proteomics.” For modified peptide enrichment, an aliquot was subjected to high-pH reversed-phase fractionation (pH 10, 8 fractions) prior to LC-MS/MS analysis.

Modified peptides were analyzed on an Orbitrap Fusion Lumos (Thermo Scientific) using targeted MS^2^ on precursor ions corresponding to CA (+132.0575 Da) and MCA (+162.0684 Da) adducts. MS^2^ spectra were manually validated for fragment ions supporting Cys359 modification.

### 2.7 Metabolomics

Metabolomics experiments were conducted according to the method with modifications (Qian et al., 2022). *C. acnes* cultures were adjusted to an initial OD_600_ of 0.2 and treated with 1 × MIC of OC for 8 h under anaerobic conditions, using 0.1% DMSO as the control. Each group comprised three biological replicates. After incubation, bacterial cells were collected by centrifugation, washed twice with ice-cold PBS, and resuspended to an OD_600_ of 0.2. Equal volumes (50 mL) of the standardized suspension were then used for metabolite extraction. Cells were disrupted in pre-cooled methanol at −80 °C via sonication (45% power, 2 s on / 3 s off, 10 min). The supernatant was collected (600 μL), and ribitol was added as an internal standard prior to vacuum evaporation to correct for technical variations during extraction and derivatization. The dried residues were derivatized with MSTFA for GC-MS analysis (Agilent 7890A) within 24 h. Metabolites were identified using the NIST and Fiehn databases, and metabolite peak areas were normalized to the internal standard (ribitol) to correct for technical variations during extraction, derivatization, and detection; no secondary normalization to cell dry weight or total protein content was performed. Differential metabolites were identified using two-tailed Student’s *t*-tests, followed by false discovery rate (FDR) correction (Benjamini–Hochberg method). Metabolites with adjusted *p*-values < 0.05 and absolute fold changes ≥ 1.5 were considered statistically significant.

### 2.8 Biofilm and B-EPS analysis

#### 2.8.1 Biofilm biomass quantification

*C. acnes* in logarithmic phase was adjusted to OD_600_ = 0.1 in BHI. One-milliliter aliquots were added to 24-well polystyrene plates and incubated anaerobically at 37 °C for 24 h. Planktonic cells were aspirated, and biofilms were washed three times with PBS. Adherent cells were fixed with methanol (15 min), stained with 0.1% (w/v) crystal violet (20 min), and rinsed with distilled water until no visible dye remained. Biofilms were air-dried for 1 h, and crystal violet was solubilized with 200 μL of 33% (v/v) acetic acid. Absorbance was measured at 570 nm (BioTek Synergy H1).

#### 2.8.2 B-EPS extraction and quantification

*C. acnes* (OD_600_ = 0.2, 6 mL) was inoculated into 90-mm polystyrene petri dishes containing OC (0, 0.5 × MIC, or 1 × MIC). Biofilms were developed anaerobically at 37 °C for 24 h without shaking. Planktonic cells were aspirated, and biofilms were gently rinsed three times with sterile PBS. Integrity was verified by inverted microscopy.

For EPS extraction, 2 mL of 50 mM sodium phosphate buffer (pH 7.0) containing Chelex-100 (5% w/v) was added. EPS was solubilized by magnetic stirring at 4 °C for 2 h. The suspension was decanted to remove resin, centrifuged (10,000 × *g*, 10 min, 4 °C), and filtered (0.22 μm). Polysaccharides were precipitated with 2.5 volumes of ice-cold ethanol at 4 °C for 2 h, collected by centrifugation (10,000 × *g*, 20 min), and redissolved in ultrapure water. The solution was dialyzed (3.5 kDa MWCO) against ultrapure water at 4 °C for 24 h (three water changes), lyophilized, and weighed. Total carbohydrate content was quantified by the phenol-sulfuric acid method using glucose as standard.

#### 2.8.3 B-EPS molecular weight and monosaccharide analysis

For monosaccharide composition, B-EPS was hydrolyzed with 2 M TFA at 110 °C for 2 h, dried, and analyzed on a Dionex ICS-5000 system equipped with a CarboPac PA20 column and pulsed amperometric detection (PAD).

For molecular weight determination, B-EPS was dissolved in 0.1 M NaNO_3_ (containing 0.05% NaN_3_), filtered (0.22 μm), and injected into a SEC-MALS system comprising a Dionex UltiMate 3000 pump, three Shodex OHpak columns (SB-805 HQ, SB-804 HQ, SB-803 HQ in series), an Optilab T-rEX refractive index detector, and a DAWN HELEOS II MALS detector (Wyatt Technology). Mobile phase: 0.1 M NaNO_3_; flow rate: 0.5 mL/min; column temperature: 35 °C. Absolute molecular weight (Mw, Mn, Mp) was calculated using ASTRA 7 software with dn/dc = 0.146 mL/g.

### 2.9 Statistical analysis

Data are expressed as mean ± SD from at least three independent biological replicates. Statistical significance was assessed by one-way ANOVA with Dunnett’s *post-hoc* test for multiple comparisons (GraphPad Prism 9.0). *P* < 0.05 was considered statistically significant. Proteomics and metabolomics data were analyzed using Perseus (v1.6.15.0) and SIMCA (v16.0.2), respectively.

## 3 Results

### 3.1 Antibacterial properties of OC and its aldehyde constituents

#### 3.1.1 Preparation and characterization of OC

**FIGURE 1:**
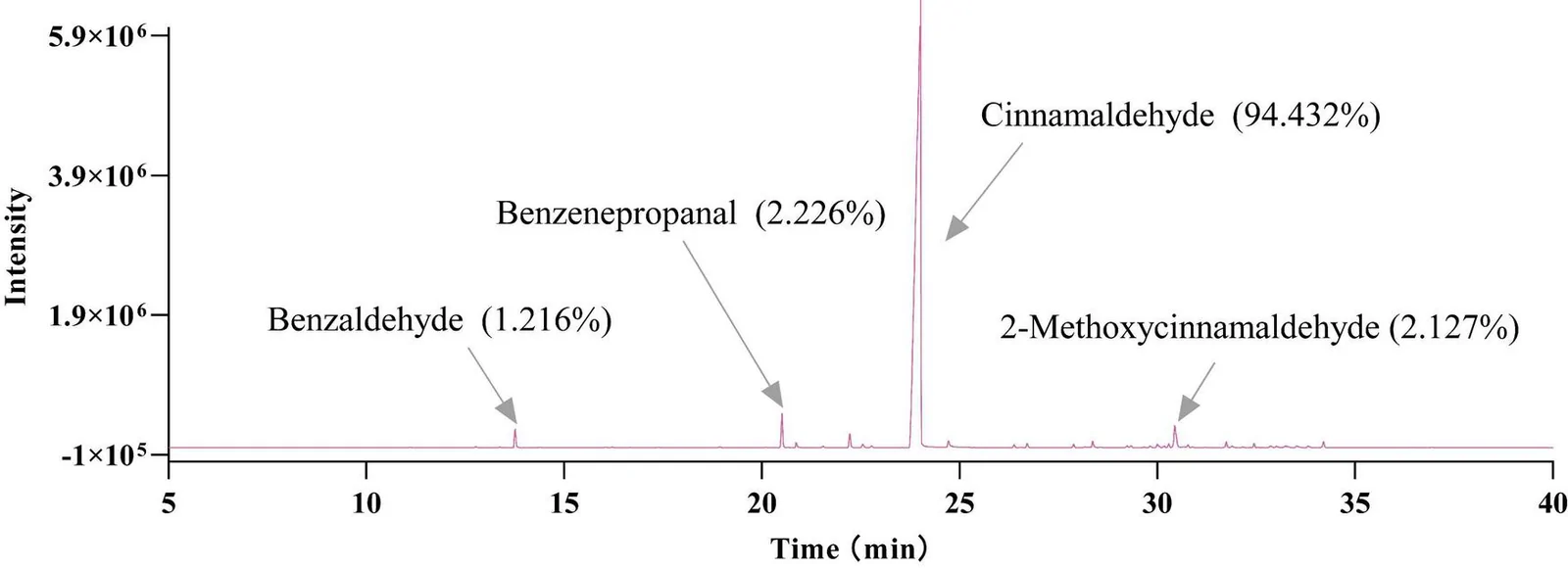
GC–MS chromatogram and chemical composition of the prepared OC.

The OC was obtained as a light-yellow, limpid oil. GC–MS analysis revealed a total cinnamaldehyde (CA) content of 94.432% (w/w), accompanied by 2.127% MCA, 2.226% benzenepropanal and 1.216% benzaldehyde (Figure 1). With CA exceeding 70%, the prepared OC complies with the specification for cinnamon oil stipulated in the 2020 Chinese Pharmacopeia (China Medical Science Press, 2020).

#### 3.1.2 Structure–activity relationship of cinnamaldehyde-derived electrophiles against *Cutibacterium acnes*

**FIGURE 2:**
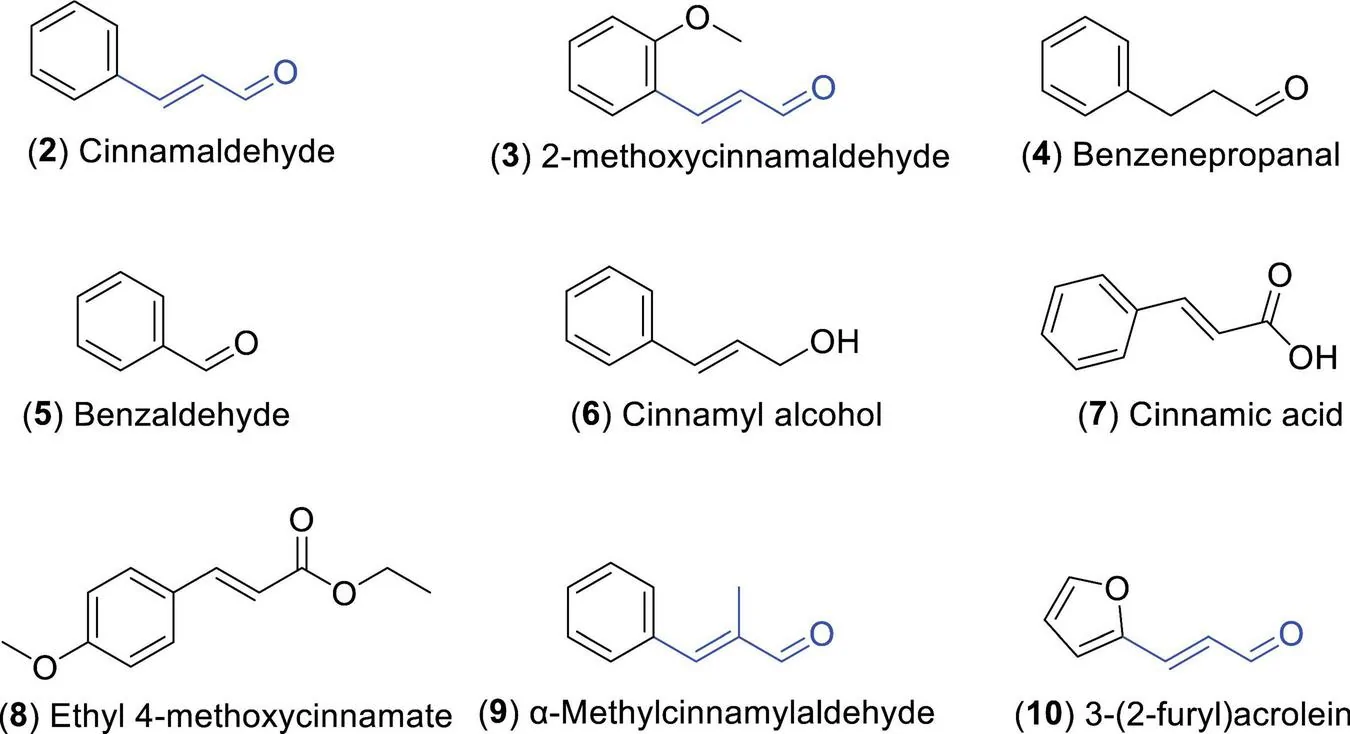
The structural formula of CA, and CA analogs.

The *in vitro* antibacterial activity of cinnamon oil (OC), cinnamaldehyde (CA), and ten CA analogs (C2–C10) (Figure 2) against *C. acnes* was systematically evaluated. As shown in Table 1, OC, CA, and MCA inhibited *C. acnes* growth with MICs of 50 or 55 μg/mL and MBCs of 150–180 μg/mL, whereas benzenepropanal and benzaldehyde exhibited markedly higher MICs (≈250–260 μg/mL). CA was identified as the principal contributor to the antibacterial activity of OC.

**TABLE 1:** MIC and MBC of test compounds against *C. acnes*.

Number	Name	MIC	MBC
1	OC	55 μg/mL	180 μg/mL
2	CA	50 μg/mL	150 μg/mL
3	MCA	50 μg/mL	150 μg/mL
4	Benzenepropanal	250 μg/mL	ND
5	Benzaldehyde	260 μg/mL	ND
6	Cinnamyl alcohol	> 1,000 μg/mL	ND
7	Cinnamic acid	> 1,000 μg/mL	ND
8	Ethyl 4-methoxycinnamate	800 μg/mL	ND
9	4-(1-methylethenyl) benzaldehyde	1,000 μg/mL	ND
10	3-(2-furyl) acrolein	600 μg/mL	ND
11	Tetracycline	1.2 μg/mL	4.8 μg/mL

Compounds devoid of an aldehyde moiety—cinnamyl alcohol, cinnamic acid, and ethyl 4-methoxycinnamate—showed negligible activity (MIC > 800 μg/mL). Likewise, analogs lacking an extended conjugated system (e.g., benzenepropanal) were poorly active. Introduction of a methyl group at the α,β-unsaturated double bond, as in 4-(1-methylethenyl)benzaldehyde, markedly attenuated activity. Collectively, the data demonstrate that the conjugated α,β-unsaturated aldehyde functionality present in CA and MCA is essential for potent anti-*C. acnes* activity, highlighting the essential role of both the aldehyde group and the conjugated double bond in antibacterial activity.

### 3.2 OC inhibits *C. acnes* adhesion to HaCaT cells

**FIGURE 3:**
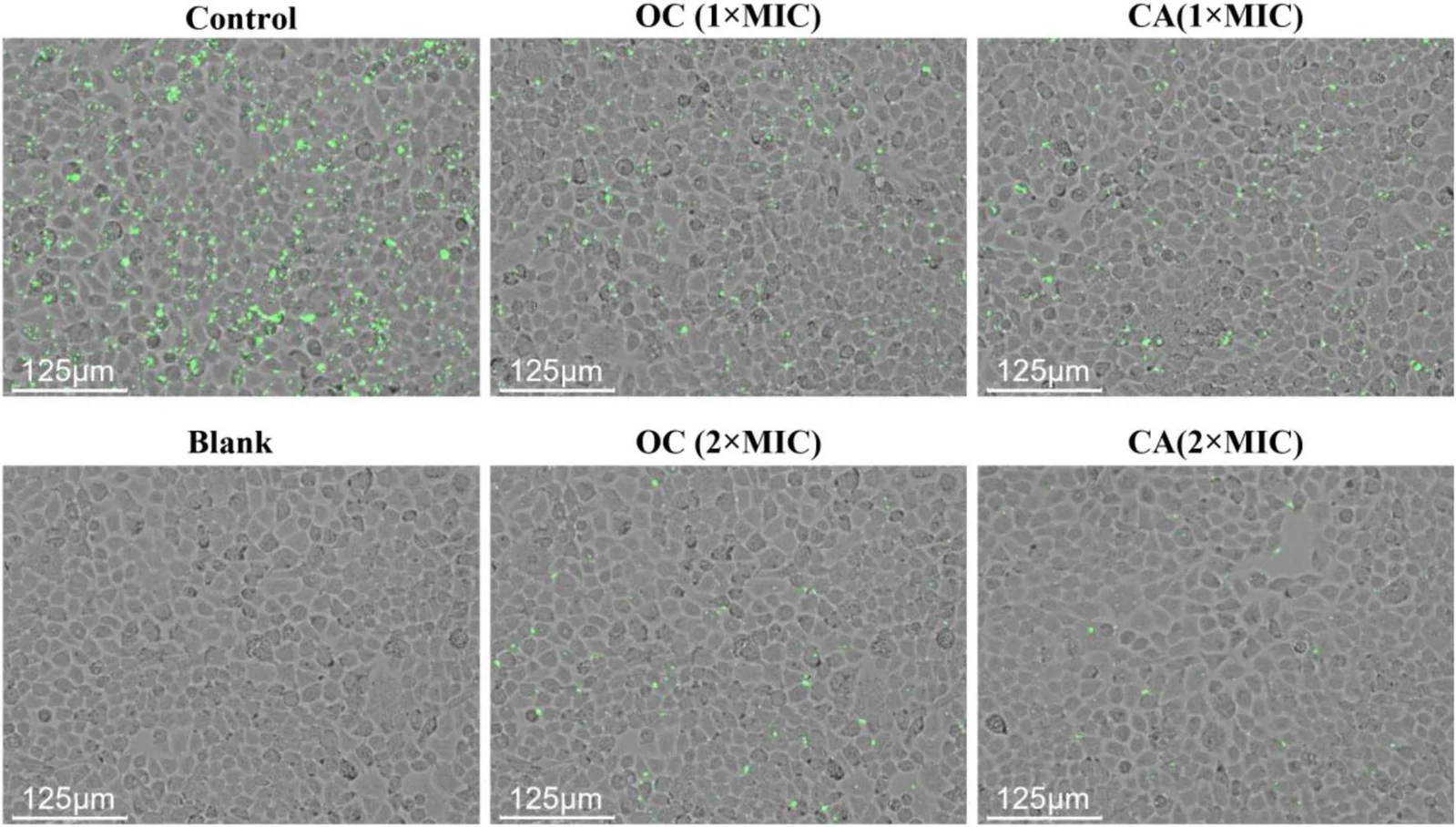
Qualitative assessment of the effect of OC and CA on *C. acnes* adhesion to HaCaT cells. Representative images captured by an integrated cell imaging system showing HaCaT cells incubated with CFDA-SE-labeled *C. acnes* in the presence of vehicle, OC (55 or 110 μg/mL), or CA (50 or 100 μg/mL).

Cutaneous colonization by *C. acnes* is initiated by bacterial adhesion to keratinocytes, which is a prerequisite for acne pathogenesis (Cavallo et al., 2022). To evaluate whether OC and CA interfere with this step, *C. acnes* was pre-treated with vehicle, OC (55 or 110 μg/mL), or CA (50 or 100 μg/mL) for 8 h under anaerobic conditions. Pre-treated bacteria were then used to infect confluent HaCaT monolayers at an MOI of 10. Qualitative assessment by integrated cell imaging (Figure 3) showed that treatment with 1 × MIC of either compound significantly reduced *C. acnes* adhesion to HaCaT cells. At 2 × MIC, adherent bacteria were markedly reduced for both compounds. These results demonstrate that OC and CA inhibit *C. acnes* adhesion to keratinocytes in a dose-dependent manner.

**FIGURE 4:**
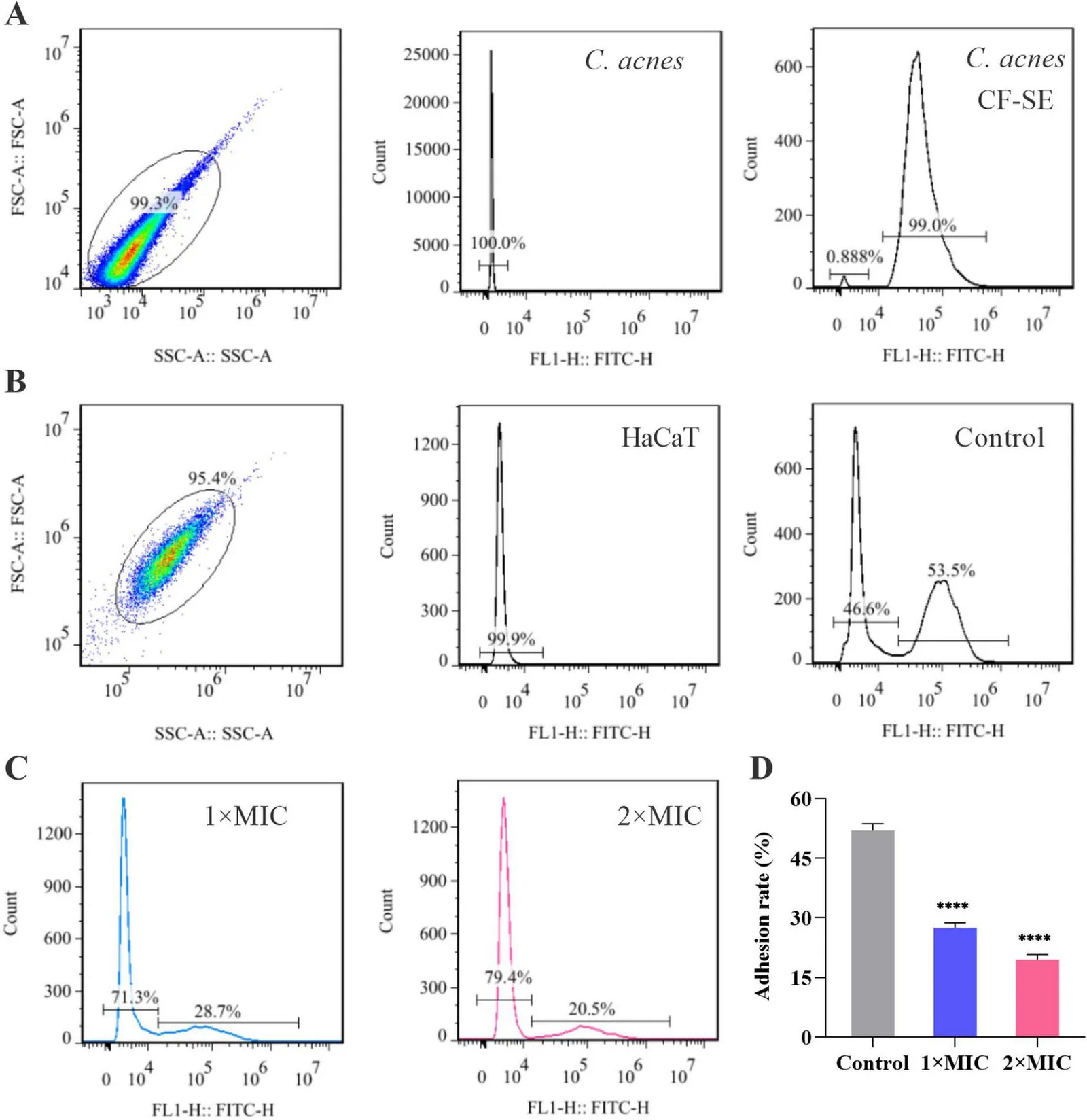
Flow-cytometric quantification of OC-mediated inhibition of *C. acnes* adhesion to HaCaT cells. **(A)** Labeling efficiency of CFDA-SE-stained *C. acnes*. **(B)** Dot plots of bacterial adhesion to vehicle-treated keratinocytes. **(C)** Adhesion profiles after exposure to OC at 1 × MIC (55 μg/mL) or 2 × MIC (110 μg/mL). **(D)** Quantitative adhesion rates (mean ± SD; *n* = 3 independent experiments). *P* < 0.01 versus vehicle control, one-way ANOVA with Dunnett’s *post-hoc* test. Asterisks were used to indicate statistically significant differences between groups (*p < 0.05, **p < 0.01, ***p < 0.001, ****p < 0.0001).

To quantitatively evaluate this effect, flow cytometry was utilized to assess OC-mediated inhibition of *C. acnes* adhesion to HaCaT cells. As shown in Figure 4A, 99.0% of bacteria incorporated the dye, confirming efficient labeling. At an MOI of 10, 53.5% of the inoculum adhered to vehicle-treated cells (Figure 4B). Exposure to 1 × MIC OC (55 μg/mL) reduced the adhesion rate to 28.7%, and 2 × MIC (110 μg/mL) further decreased it to 20.5% (Figure 4C). Thus, OC conferred approximately 50% and 62% inhibition at 1 × and 2 × MIC, respectively (*P* < 0.01 versus vehicle control, one-way ANOVA with Dunnett’s *post-hoc* test; Figure 4D). These data establish that OC dose-dependently blocks *C. acnes* adhesion to HaCaT cells.

### 3.3 Proteomics

**FIGURE 5:**
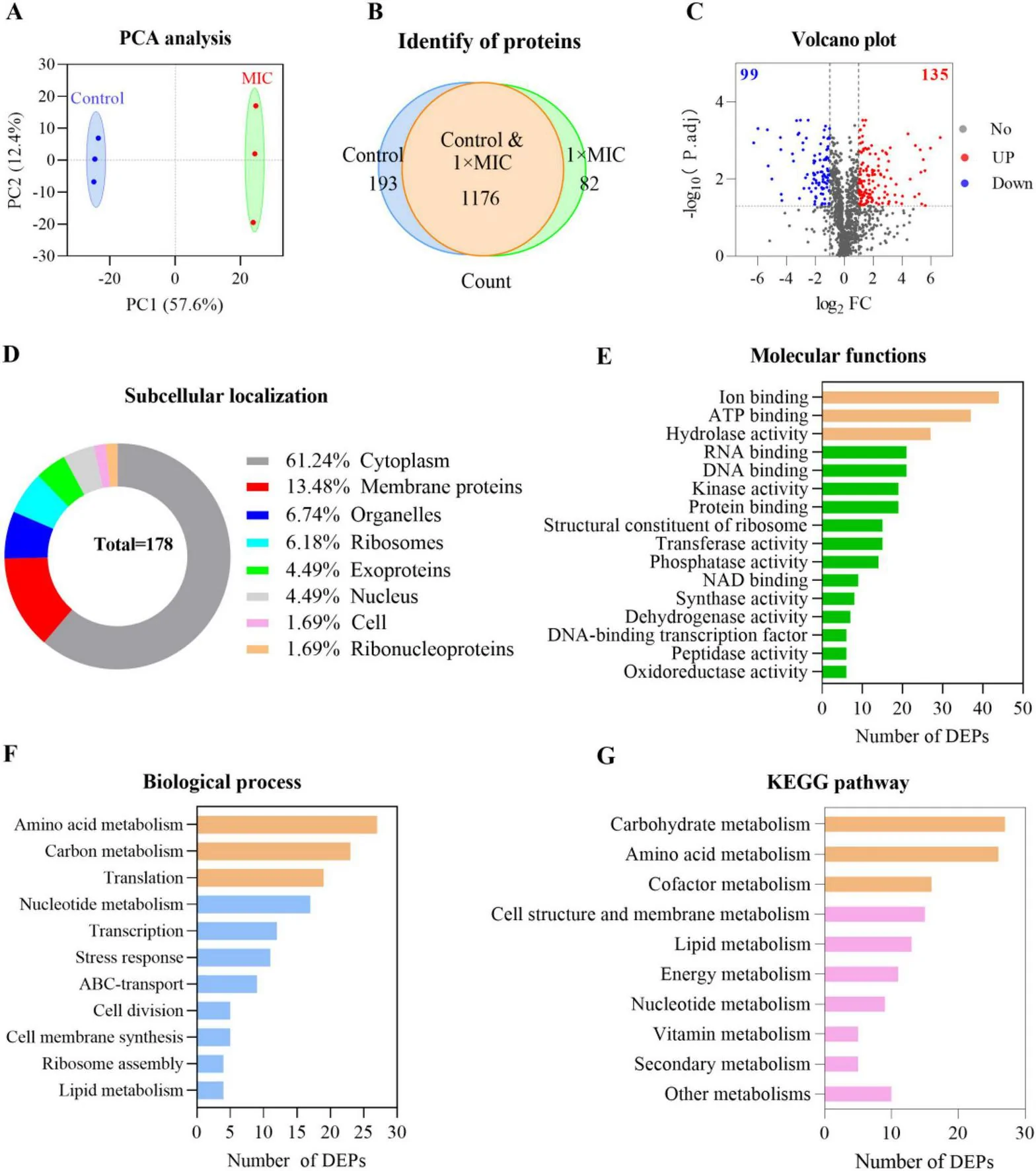
Quantitative proteome map of *C. acnes* exposed to OC. **(A)** PCA of global protein-expression profiles. **(B)** Venn diagram of proteins identified in control and OC-treated conditions. **(C)** Volcano plots highlighting differentially expressed proteins (DEPs). **(D)** Subcellular localization of DEPs. **(E)** GO molecular-function classification. **(F)** Biological processes enriched among DEPs. **(G)** KEGG pathway enrichment of the top perturbed metabolic routes.

Label-free quantitative proteomics was employed to map the intracellular response of *C. acnes* to 1 × MIC OC (Figure 5). Principal-component analysis separated treated and control proteomes along PC1 and PC2, which together explained 70% of the variance (Figure 5A). A total of 1,176 proteins (UniProt *C. acnes* database, downloaded 5 May 2024) were commonly identified in both conditions by Proteome Discoverer 2.2.0.388 (Thermo Scientific) (Figure 5B). Volcano filtering (*P* < 0.05) yielded 234 differentially expressed proteins (DEPs): 135 up-regulated and 99 downregulated (Figure 5C). Subcellular localization assigned 178 DEPs to all major compartments; 61.24% were cytoplasm, 13.48% membrane proteins, 6.74% organelles, 6.18% ribosomes, 4.49% exoproteins, 4.49% nucleus, 1.69% cell wall, and 1.69% as ribonucleoproteins (Figure 5D). Gene ontology (GO) classification revealed dominant molecular functions of metal-ion binding, ATP binding, hydrolase, RNA/DNA binding and kinase activities (Figure 5E). Biological-process terms were enriched for amino-acid, carbon and nucleotide metabolism, translation, transcription, stress response, ABC transport, cell division, membrane biogenesis, ribosome assembly and lipid metabolism (Figure 5F). KEGG pathway enrichment (top 10) highlighted carbohydrate, amino-acid and cofactor metabolism as the most significantly perturbed routes, followed by lipid, energy, nucleotide, vitamin and secondary metabolism (Figure 5G). Collectively, OC appears to disable *C. acnes* by simultaneous disruption of energy homeostasis (carbohydrate metabolism), protein biosynthesis (amino-acid metabolism) and enzymatic capacity (cofactor and ion-dependent catalysis), indicating multi-target synergy rather than single-protein inhibition.

### 3.4 Analysis of chemical modification of proteins

**FIGURE 6:**
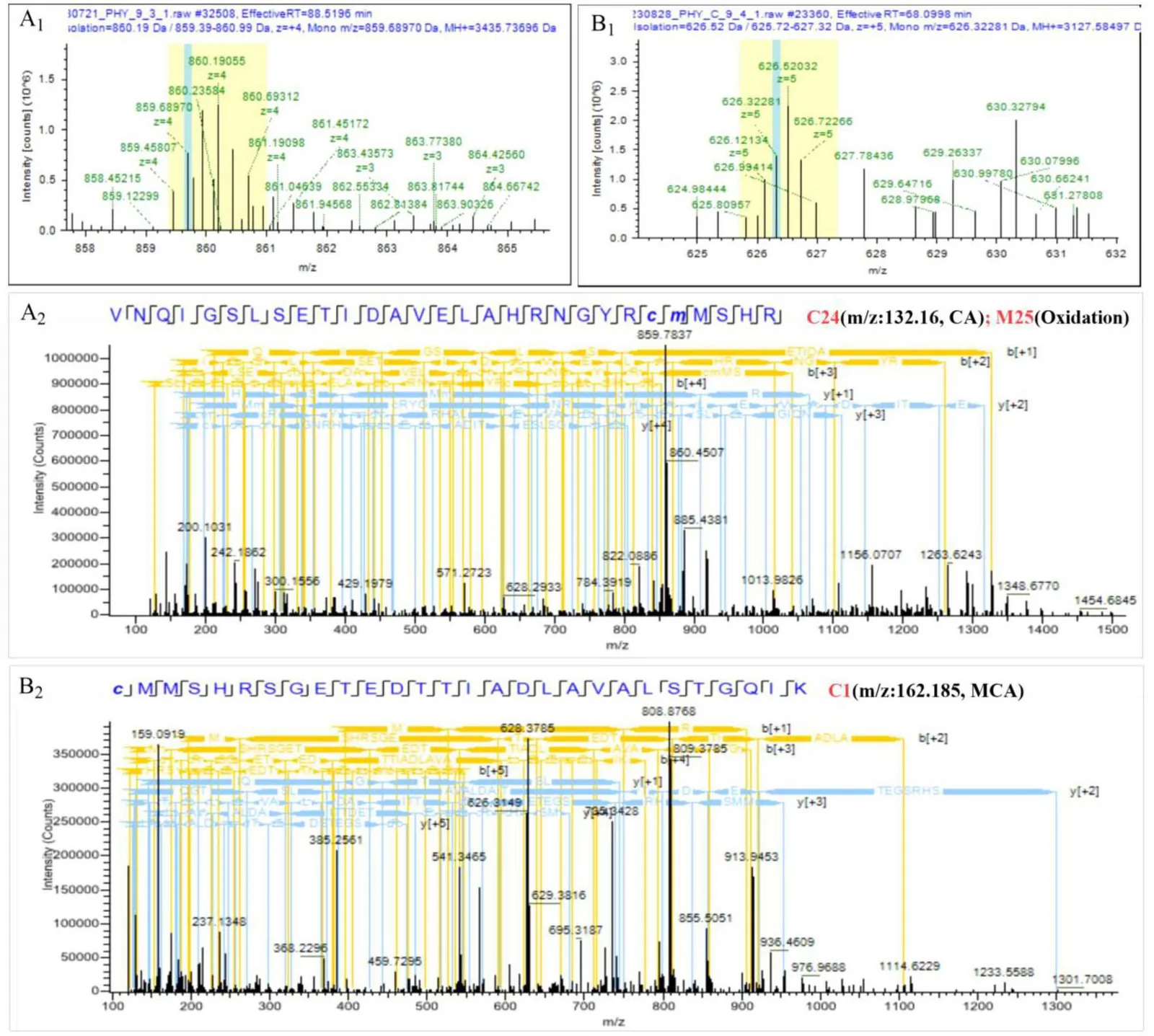
MS/MS spectra of enolase peptides carrying OC-derived covalent modifications **(A)** VNQIGSLSETIDAVELAHRNGYRCMMSHR: CA adduct (+132.05 Da) at Cys359 (C24 of the peptide). **(B)** CMMSHRSGETEDTTIADLAVALSTGQIK: MCA adduct (+162.05 Da) at Cys359 (C1 of the peptide).

Modified proteomics techniques have emerged as a powerful tool for investigating protein modifications and inhibitors, enabling effective identification of active and binding sites (Cuevas-Cruz et al., 2023). Chemical proteomics pinpointed enolase (A0AA44ZFR3) as a covalent target of OC in *C. acnes*. LC–MS/MS revealed mono-adducts of +132.0575 Da and +162.0684 Da exclusively at Cys359, corresponding to CA and MCA, respectively; no modification was detected in vehicle-treated cells Figure 6 (A1, A2, B1, B2). No such modifications were observed in the control group under the same experimental conditions. These adduct masses are consistent with addition of CA and MCA to the cysteine thiol. Based on the known reactivity of α,β-unsaturated aldehydes with cysteine thiol groups (−SH), it is postulated that CA and MCA form adducts with Cys359 of ENO. This finding confirms the covalent modification of ENO by CA and MCA.

Enolase is the glycolytic enzyme that interconverts 2-phosphoglycerate (2-PG) and phosphoenolpyruvate (PEP). Its Cys359 lies within the C-terminal TIM-barrel that harbors all catalytic loops but is not itself a catalytic residue. Nevertheless, rigid CA/MCA conjugates introduced here are expected to sterically distort the barrel and impair the conformational flexibility required for proton abstraction by Lys386/His155 and for Mg^2+^-mediated intermediate stabilization (Boël et al., 2004). Such allosteric blockage would lower intracellular PEP levels and reduce ATP yield, potentially compromising energy-dependent processes including cell-wall biosynthesis and surface adhesin expression, thereby contributing to the observed inhibition of *C. acnes* adhesion to host cells.

### 3.5 Covalent interaction between CA and coenzyme A

**FIGURE 7:**
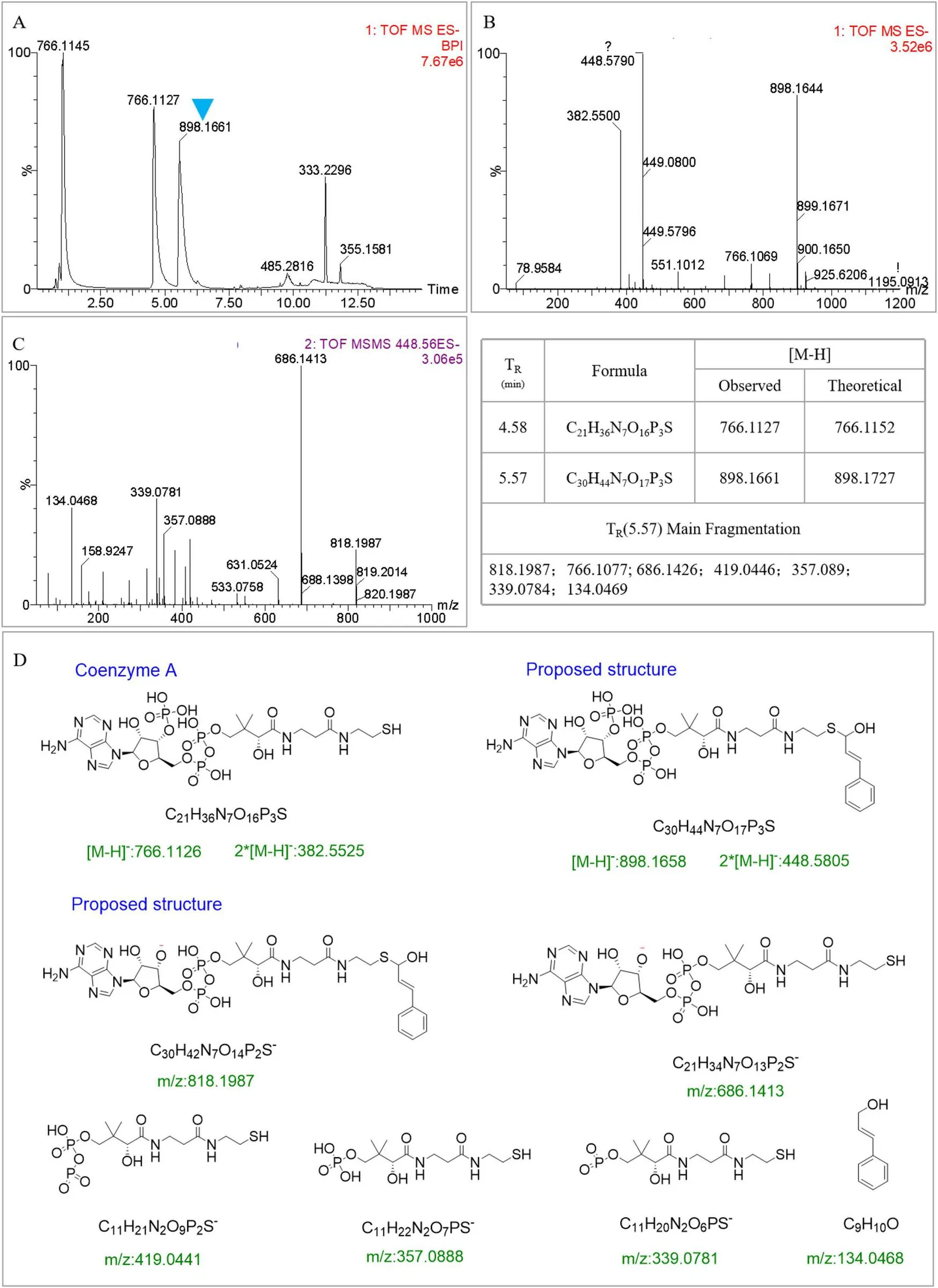
The reaction products of CA and CoA. **(A)** Total ion chromatogram. **(B)** Primary mass spectrum at retention time (5.57 min). **(C)** Secondary mass spectrum at retention time (5.57 min). **(D)** Summary of fragment ions.

Previous studies have demonstrated that CA covalently modifies cysteine residues in ENO. Given that coenzyme A (CoA) contains a cysteine moiety within its structure, we hypothesized that CA would also form a covalent bond with CoA via a similar mechanism. To investigate this, we incubated CA with CoA in PBS at 37 °C for 2 h and analyzed the resulting adducts using UPLC-MS/MS. In the total ion chromatogram, the [M−H] ion of CoA was detected at m/z 766.1445 and 766.127 (Figure 7A). A distinct peak was observed at a retention time (RT) of 5.57 min, with an m/z value of 898.1661, corresponding to the [M−H] ion of the CA–CoA adduct. The mass spectrum at this retention time displayed three major peaks at m/z 898.1644 ([M−H] of the adduct), 448.579 ([M−2H]^2^ of the adduct), and 382.55 ([M−2H]^2^ of free CoA) (Figure 7B).

Further fragmentation analysis revealed characteristic product ions at m/z 818.1987, 686.1413, 419.0441, 357.0888, 339.0781, and 134.0468 (Figure 7C), supporting the proposed covalent adduct structure (Figure 7D). To assess the stoichiometry, reactions were performed at CA:CoA molar ratios of 1:0.5, 1:1, and 1:2. In all cases, the predominant ion was observed at m/z 898.1661 ([M−H]) or m/z 448.579 ([M−2H]^2^), with no evidence of doubly modified CoA even at excess CA. These results are consistent with the formation of a 1:1 CA–CoA adduct, likely via thiohemiacetal formation at the aldehyde group, yielding a stable thioether linkage (Peng et al., 2026). This stability may be attributed to steric hindrance, which confers structural rigidity to the thiohemiacetal intermediate.

### 3.6 OC rewires *C. acnes* glucose metabolism via covalent targeting of ENO and CoA

**FIGURE 8:**
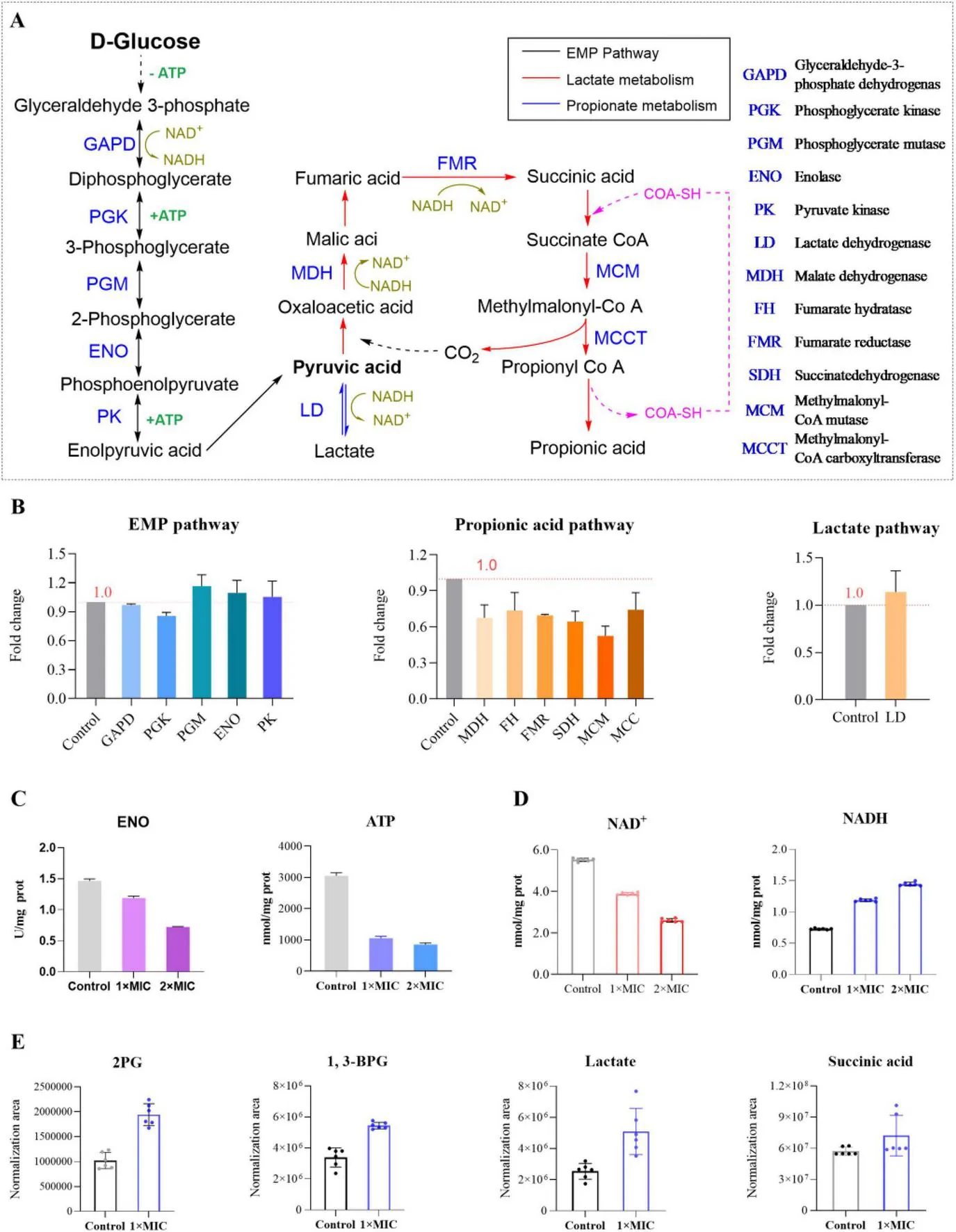
OC rewires *C. acnes* glucose metabolism via covalent targeting of ENO and CoA. **(A)** Scheme of the EMP–propionate pathway and lactate shunt. **(B)** Proteomic analysis of core enzymes in glycolysis, propionate and lactate branches. **(C)** ENO activity and ATP in CA-treated versus control *C. acnes*. **(D)** Intracellular NAD^+^ and NADH levels. **(E)** GC-MS profiling of 2PG, 1,3-BPG, lactate and succinate.

Under anaerobic conditions, *C. acnes* metabolizes glucose to pyruvate via the Embden–Meyerhof–Parnas (EMP) pathway, yielding two ATP molecules and reducing two NAD^+^ to NADH. Pyruvate is subsequently converted to propionic acid, enabling ATP generation and redox homeostasis (Figure 8A). Under stress conditions, pyruvate may alternatively be reduced to lactate. Enolase (ENO) catalyzes the interconversion of 2-phosphoglycerate (2PG) to phosphoenolpyruvate (PEP) in the ninth step of the EMP pathway, serving a critical role in both glycolysis and gluconeogenesis. Coenzyme A (CoA) participates in propionic acid metabolism by reacting with succinate to form succinyl-CoA, a reaction catalyzed by succinate dehydrogenase (SDH). This step facilitates propionic acid synthesis and regenerates CoA for subsequent metabolic cycles. Thus, both ENO and CoA are essential for maintaining glucose metabolism in *C. acnes*.

This study investigated the metabolic consequences of OC exposure in *C. acnes*, wherein the active constituent CA (94.432% of the total OC composition) targets ENO and CoA. Compared to untreated controls, exposure to OC at 1 × MIC significantly upregulated the expression of key glycolytic enzymes, including glyceraldehyde-3-phosphate dehydrogenase (GAPD), phosphoglycerate kinase (PGK), phosphoglycerate mutase (PGM), ENO, and pyruvate kinase (PK). In contrast, enzymes involved in propionic acid metabolism—such as malate dehydrogenase (MDH), fumarate hydratase (FH), fumarate reductase (FMR), SDH, methylmalonyl-CoA mutase (MCM), and methylmalonyl-CoA decarboxylase (MCCT)—were downregulated, while lactate dehydrogenase (LDH) expression was induced (Figure 8B).

Under OC stress, intracellular glycolytic flux increased, resulting in elevated NADH accumulation (Figure 8D). However, the CA-induced covalent modification of ENO led to a concentration-dependent decrease in its enzymatic activity, impairing the conversion of 2PG to PEP. This blockade caused intracellular accumulation of 2PG and 1,3-bisphosphoglycerate (1,3-BPG), ultimately disrupting pyruvate production. Additionally, the CA-mediated sequestration of intracellular CoA impaired the succinate–propionate pathway, leading to succinate accumulation (Figure 8E). This metabolic bottleneck inhibited NADH reoxidation during the second phase of anaerobic respiration.

Consequently, *C. acnes* shifted its metabolic flux toward lactate fermentation, as evidenced by upregulated LDH expression and increased lactate levels. OC treatment resulted in a marked rise in intracellular NADH and a corresponding decline in NAD^+^, accompanied by reduced ATP production (Figure 8C). These findings collectively demonstrate that OC disrupts glucose metabolism in *C. acnes* via its active constituent CA by targeting both ENO and CoA, redirecting carbon flux from propionate to lactate and compromising energy generation and redox balance.

### 3.7 OC affects carbohydrate metabolism and B-EPS biosynthesis in *C. acnes*

**FIGURE 9:**
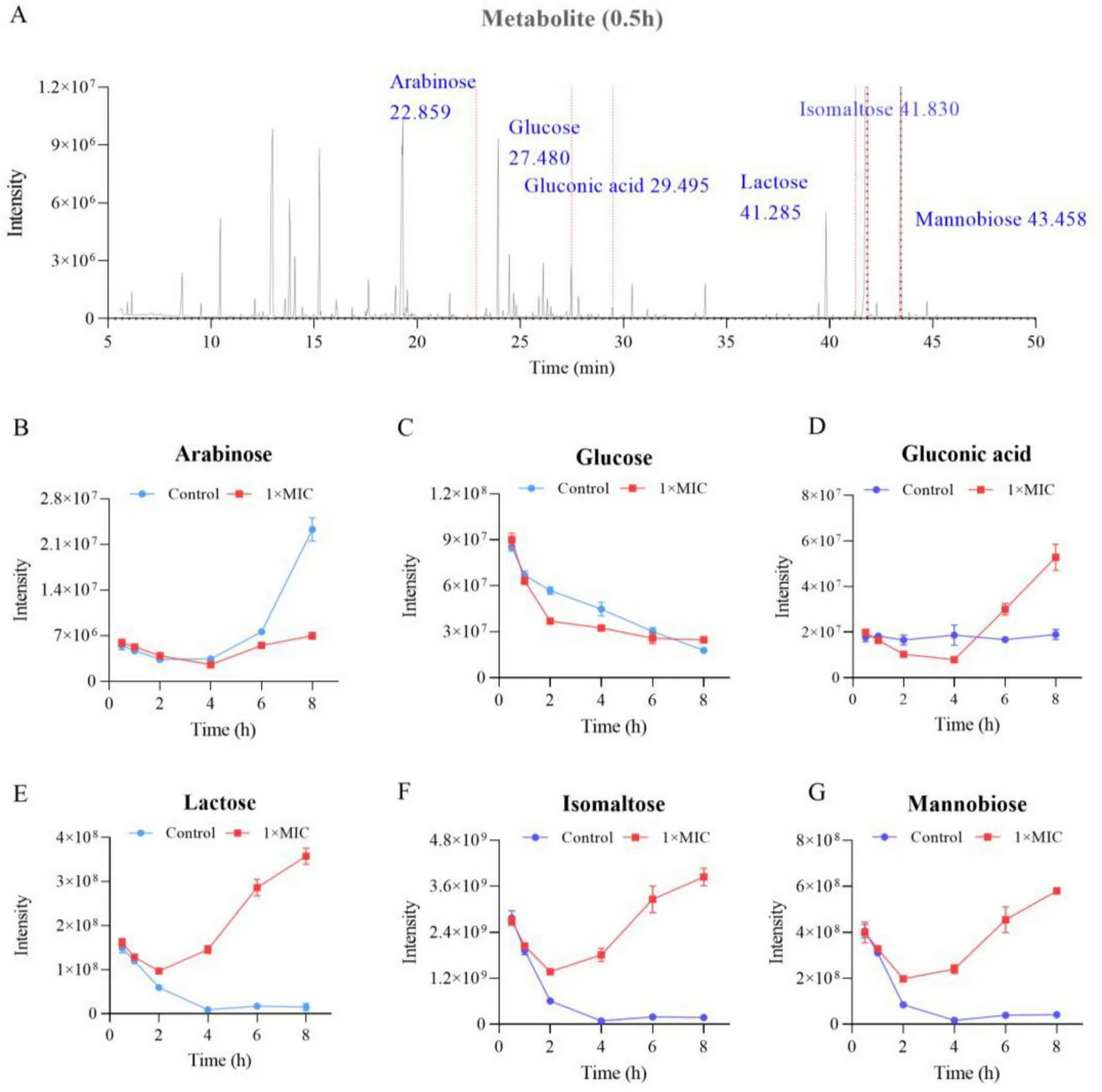
OC disrupts oligosaccharide homeostasis in *C. acnes* (GC-MS profiling). **(A)** Representative total-ion chromatogram (0.5 h). **(B–G)** Time-dependent intracellular abundances of arabinose, glucose, gluconic acid, lactose, isomaltose and mannobiose, respectively. Data represent mean ± SD; *n* = 3.

To visualize how OC rewires carbohydrate flux, we monitored intracellular levels of arabinose, glucose, gluconic acid, lactose, isomaltose and mannobiose by GC-MS during 8 h exposure to 1 × MIC OC (Figure 9A). Arabinose remained constant in OC-treated cells, whereas it steadily accumulated in controls after 4 h (Figure 9B). Glucose levels declined sharply within 2 h under OC stress and subsequently decreased at a markedly reduced rate from 2 to 8 h; in contrast, control cells consumed glucose continuously (Figure 9C). Gluconic acid decreased during 0.5–4 h in OC-exposed cells, then re-accumulated at 4–8 h, while controls showed no fluctuation (Figure 9D). Lactose, isomaltose and mannobiose dropped rapidly in controls (0.5–4 h) and approached zero by 8 h; under OC, the initial decline was slower, followed by sustained intracellular accumulation from 2 h onward (Figure 9E–G).

These kinetic distortions reflect the metabolic bottleneck imposed by OC. By covalently inhibiting ENO and CoA within 2 h, OC halts glycolytic flux and forces upstream carbohydrates to stall or reroute. Arabinose, normally channeled into the pentose-phosphate pathway to support NADPH and nucleotide synthesis during active growth (Tang et al., 2021), accumulates in controls reflecting active biomass expansion, whereas OC-treated cells maintain basal levels due to suppressed metabolic demand. Gluconic acid—an intermediate of glucose oxidation—transiently decreased and then re-accumulated, reflecting altered flux through oxidative glucose metabolism pathways (Lee et al., 2006). Accumulation of disaccharides reveals a broader breakdown of polysaccharide turnover and biofilm precursor synthesis, compromising energy extraction, protective matrix production and cell-surface adhesion (Mareya et al., 2020). Collectively, the data show that OC-driven targeting of ENO and CoA does not merely slow glucose catabolism; it deranges the entire oligosaccharide network of *C. acnes*.

**FIGURE 10:**
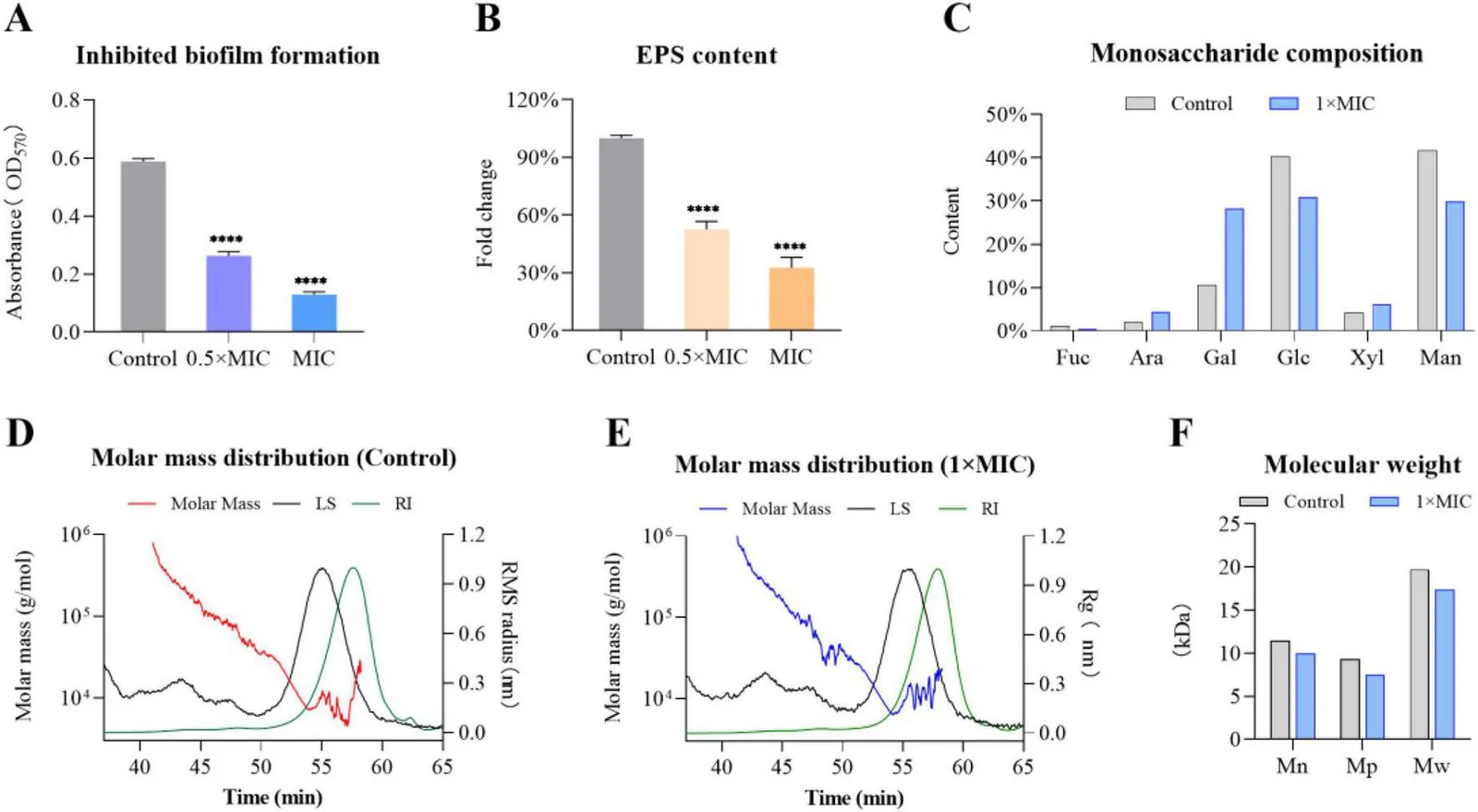
OC impairs B-EPS biosynthesis and polymer architecture in *C. acnes*. **(A)** Biofilm biomass quantified by crystal-violet assay. **(B)** Total B-EPS yield determined with the phenol–sulfuric acid method. **(C)** Monosaccharide composition of B-EPS analyzed by high-performance anion-exchange chromatography (ICS-5000). **(D)** SEC-MALLS chromatogram of control B-EPS. **(E)** SEC-MALLS chromatogram of B-EPS from OC-treated cells. **(F)** Comparative summary of Mn, Mw, and Mp values. Asterisks were used to indicate statistically significant differences between groups (*p < 0.05, **p < 0.01, ***p < 0.001, ****p < 0.0001).

Extracellular polysaccharides constitute the scaffold of microbial biofilms and confer physical integrity as well as resistance to antimicrobials and host immunity (Bampidis et al., 2022). To determine whether OC compromises this protective matrix, we quantified biofilm biomass and biofilm exopolysaccharides (B-EPS) production in *C. acnes* exposed to 0.5 × MIC and 1 × MIC of OC. Biofilm biomass decreased by 50% at 0.5 × MIC and by 75% at 1 × MIC after 8 h, indicating a dose-dependent inhibition of biofilm formation (Figure 10A). Concomitantly, total B-EPS dropped by 48.4% and 67.4% under the same conditions (Figure 10B), confirming that OC targets the B-EPS itself rather than merely preventing cell adhesion. Monosaccharide analysis revealed that control B-EPS is rich in hexoses (galactose, glucose, mannose, fucose) with minor pentoses (arabinose, xylose) (Figure 10C). Size-exclusion chromatography further showed a significant downward shift in molecular-weight distribution: number-average (Mn), weight-average (Mw), and peak (Mp) values fell from 11.4, 19.7, and 9.3 kDa (control) to 9.9, 17.3, and 7.5 kDa (OC-treated), respectively (Figures 10D–F). The shorter B-EPS chains are predicted to form a less cohesive three-dimensional lattice, weakening the structural integrity required for mature biofilm formation (Iaconis et al., 2024). By interfering with central carbon flux via ENO and CoA inhibition, OC deprives *C. acnes* of the precursor supply necessary for high-molecular-weight B-EPS synthesis. The resulting matrix deficiency reduces biofilm thickness, lowers mechanical stability, and ultimately impairs bacterial adherence to host surfaces—thereby undermining the protective niche that confers antibiotic tolerance and immune evasion.

## 4 Discussion and conclusion

This study, through antimicrobial phenotypic analysis and mass spectrometry–based target identification, demonstrates that the major active constituent of OC, CA, remodels the central carbon metabolism of *C. acnes* via covalent modification of ENO and CoA. Direct experimental evidence indicates that the α,β-unsaturated aldehyde moiety of CA forms a thiohemiacetal adduct with Cys359 of ENO, which may restrict the conformational dynamics of the TIM-barrel domain through steric hindrance, thereby blocking the conversion of 2-PG to PEP and causing a concentration-dependent decrease in ENO enzymatic activity (Balducci et al., 2023); concurrently, CA forms a stable thiohemiacetal with the reactive thiol terminus of CoA, disrupting acyl transfer reactions (Norton et al., 2012). Proteomic data further support this model: OC treatment significantly upregulates proximal glycolytic enzymes (GAPD, PGK, PGM, PK) while suppressing downstream propionic acid pathway proteins (MDH, FH, FMR, SDH, MCM, MCCT). These metabolic perturbations are closely associated with the antimicrobial phenotype of OC, including depletion of ATP, NAD^+^, and biosynthetic precursors, reduced EPS production, decreased biofilm formation, and a concentration-dependent decline in *C. acnes* adhesion to HaCaT cells.

Previous studies on the antimicrobial effects of OC/CA have revealed multiple mechanisms in various pathogenic bacteria, including induction of oxidative stress and acid stress (Amalaradjou and Venkitanarayanan, 2011), disruption of membrane structural integrity (Gill and Holley, 2006), inhibition of FtsZ to block cell division (Domadia et al., 2007), and interference with quorum-sensing systems to reduce virulence factor expression (Brackman et al., 2011), collectively characterizing the broad-spectrum toxic stress effects of aldehyde compounds on bacteria (Burt, 2004). Notably, the α,β-unsaturated aldehyde moiety of CA confers dual electrophilicity (at the carbonyl carbon and the β-carbon), enabling it to undergo thioacetal reactions with protein thiols (Peng et al., 2026); however, the direct molecular link between this covalent modification potential and the antimicrobial phenotype has not been established. In particular, the mechanism by which CA reprograms bacterial energy metabolism through covalent modification of central carbon metabolic enzymes, thereby inhibiting biofilm formation and host adhesion, has hitherto lacked high-resolution target identification and functional validation at the metabolomic level. This study, by integrating proteomics, metabolomics, and antimicrobial phenotypic analysis, reveals an anti-adhesion pathway centered on central metabolic reprogramming.

Unlike the reversible binding of non-covalent drugs, the covalent adducts formed between CA and ENO Cys359 or CoA thiol groups persistently block target function until protein regeneration, thereby effectively preventing target reactivation (Kim et al., 2021). From the metabolic network perspective, ENO inhibition creates a bottleneck at the 2-PG/PEP node, which not only leads to accumulation of upstream glycolytic intermediates but, more critically, deprives the supply of pyruvate and its downstream products—both serving as core carbon sources for nucleotide-sugar precursors and membrane lipid components required for EPS synthesis (López-Diez et al., 2021). Meanwhile, CoA pathway blockage stalls the conversion of succinate to propionate, impedes NADH reoxidation, and forces the cell to redirect metabolic flux toward lactate fermentation to maintain redox balance (Kim et al., 2023). This dual insult—energy crisis coupled with biosynthetic precursor shortage—directly explains why OC treatment results in shortened EPS chain length, hexose deficiency, and reduced biofilm biomass (Netrusov et al., 2023). It should be explicitly noted that the diversion of carbon flux toward lactate fermentation to compensate for energy deficit, and the direct restriction of EPS precursor supply by metabolic flux redistribution, are mechanistic inferences proposed based on proteomic changes and metabolite accumulation patterns, and require further validation. Future studies will employ metabolic rescue experiments, including exogenous ATP supplementation and specific fatty acid repletion, combined with genetic approaches such as site-directed mutagenesis of ENO, to provide definitive evidence for the causal relationship between metabolic defects and the adhesion phenotype.

Beyond its canonical glycolytic function, ENO serves as a surface-exposed plasminogen (Plg) receptor and extracellular matrix-binding protein with moonlighting activities in various pathogens, including *Staphylococcus aureus*, *Streptococcus pneumoniae*, *Streptococcus suis* (Li et al., 2022), *Lactobacillus plantarum* (Glenting et al., 2013), *Mycoplasma bovis* (Chen R. et al., 2022), *M. fermentans* (Chen et al., 2019), and *Trichomonas vaginalis* (Alderete and Neace, 2013). Surface ENO captures host Plg and extracellular RNA to activate the fibrinolytic cascade, facilitating bacterial traversal of endothelial or epithelial barriers (Figueiredo et al., 2015); in *S. aureus*, ENO can also bind laminin to promote dissemination within the extracellular matrix (Hemmadi et al., 2022). We speculate that covalent modification of ENO Cys359 by CA may exert dual effects: on one hand, intracellular ENO inhibition disrupts energy metabolism and depletes EPS precursors, causing defects in biofilm matrix synthesis; on the other hand, if CA modifies ENO localized on the bacterial surface, it may directly impair interactions with host Plg or the extracellular matrix, thereby attenuating bacterial adhesion independently of metabolic effects. It should be emphasized that the direct contribution of impaired surface ENO moonlighting function to reduced adhesion has not been experimentally validated; future studies may address this through surface protein biotinylation or Plg binding assays.

In this study, *C. acnes* was pretreated with OC for 8 h, after which residual drug was removed before co-incubation with HaCaT cells to assess bacterial adhesion. This strategy aims to simulate a physiologically relevant scenario in which bacteria are exposed to an antimicrobial agent prior to host contact, focusing on how OC indirectly affects subsequent adhesion behavior by remodeling the bacteria’s intrinsic properties, thereby providing a research framework for understanding how drug exposure modulates the pathogenic potential of *C. acnes*. Building upon this foundation, future studies may employ temporal controls, including co-administration and post-adhesion administration, to further elucidate the stage-specific effects of OC at different phases of the adhesion kinetics.

Although this study identified the covalent modification site at ENO Cys359 by high-confidence MS/MS (site localization probability > 0.95) and provided supporting evidence through decreased enzymatic activity and metabolic disruption, thereby preliminarily supporting ENO as a candidate target of OC, the modification occupancy within the total ENO protein pool remains to be determined. Subsequent studies may employ targeted multiple reaction monitoring MRM to quantify modification occupancy, combined with site-directed mutagenesis of ENO, to establish the causal contribution of covalent modification to metabolic disruption.

Of greater translational significance, based on the action mode of CA in covalently modifying metabolic nodes (ENO/CoA), the development of selective covalent inhibitors targeting pathogen-specific cysteines using α,β-unsaturated aldehydes as lead scaffolds holds promise for providing novel candidates for topical anti-acne therapy.

## Data Availability

The original contributions presented in this study are included in this article/supplementary material, further inquiries can be directed to the corresponding author.
